# The Influence of Young Age on Difficulties in the Surgical Resection of Carotid Body Tumors

**DOI:** 10.3390/cancers13184565

**Published:** 2021-09-11

**Authors:** Kartsunori Katagiri, Kiyoto Shiga, Aya Ikeda, Daisuke Saito, Shin-ichi Oikawa, Kodai Tsuchida, Jun Miyaguchi, Takahiro Kusaka, Akio Tamura, Manabu Nakayama, Mitsuru Izumisawa, Kenji Yoshida, Kuniaki Ogasawara, Fumiaki Takahashi

**Affiliations:** 1 Department of Head and Neck Surgery, Iwate Medical University School of Medicine, Yahaba, Morioka 028-3695, Iwate, Japan; kkatagir@iwate-med.ac.jp (K.K.); aikeda@iwate-med.ac.jp (A.I.); daisaito@iwate-med.ac.jp (D.S.); soikawa@iwate-med.ac.jp (S.-i.O.); ktsuchid@iwate-med.ac.jp (K.T.); miyamj@iwate-med.ac.jp (J.M.); tkusaka@iwate-med.ac.jp (T.K.); 2 Department of Radiology, Iwate Medical University School of Medicine, Yahaba, Morioka 028-3695, Iwate, Japan; akahane@iwate-med.ac.jp (A.T.); mnakayama@iwate-med.ac.jp (M.N.); mizumisa@iwate-med.ac.jp (M.I.); 3 Department of Neurosurgery, Iwate Medical University School of Medicine, Yahaba, Morioka 028-3695, Iwate, Japan; kenyoshi@iwate-med.ac.jp (K.Y.); kuogasa@iwate-med.ac.jp (K.O.); 4 Division of Medical Engineering, Department of Information Science, Iwate Medical University School of Medicine, Yahaba, Morioka 028-3695, Iwate, Japan; ftakahas@iwate-med.ac.jp

**Keywords:** carotid body tumor, surgical resection, carotid artery reconstruction, post-operative complication, younger age

## Abstract

**Simple Summary:**

The aim of this study was to reveal the factors affecting the complexity and difficulties in performing surgery to resect carotid body tumors (CBTs). We analyzed 20 patients with 21 CBTs. We used the “same day surgery” procedure, including preoperative embolization of the feeding arteries in the morning and resection surgery in the afternoon of the same day. Four patients underwent resection of the carotid artery, followed by reconstruction. These four patients were between 18 to 23 years of age at the time of surgery. The mean blood loss and operative time in these patients differed significantly from those in older patients. These results indicated that young age may influence the difficulties faced in CBT surgery, resulting in an increased risk of carotid artery resection. The results obtained from our study could help surgeons safely and effectively perform resection surgery for CBTs.

**Abstract:**

This study evaluated patient characteristics that affect the complexity and difficulties of performing surgery to resect carotid body tumors (CBTs). We retrospectively reviewed the medical records of 20 patients with 21 CBTs who were enrolled in the study. The median patient age was 46 years and the mean tumor diameter was 37.6 mm. The mean blood loss and operative time were 40.3 mL and 183 min, respectively. Four patients underwent resection of the carotid artery followed by reconstruction. These four patients were between 18 to 23 years of age at the time of surgery. The mean blood loss and operative time in these patients were 166 mL and 394 min, respectively, which differed significantly from those of older patients. Therefore, young age influenced the difficulties faced in surgical resection of CBT, with an increased risk of blood loss and carotid artery resection.

## 1. Introduction

Carotid body tumors (CBTs) are rare neoplasms that originate from the paraganglion cells in the carotid body. It is known that CBTs are surrounded by many tumor-feeding arteries, resulting in a rich vascular supply [1]. Molecular biology research has revealed various types of gene alterations in CBTs, including variants of the succinate dehydrogenase (SDH) gene family [2,3,4]. “Hereditary paraganglioma-pheochromocytoma syndrome” is a rare condition representing patients with familial pheochromocytoma and/or paraganglioma; however, this also applies to CBT patients [5,6,7]. In 2017, CBT was recognized as a malignant tumor in the World Health Organization (WHO) tumor classification system [8]. Head-and-neck surgeons must consider surgical resection of CBT once a patient is referred to the hospital. However, the rarity and vascular-rich features of this tumor can make surgical resection very difficult in some cases. Although three meta-analyses of preoperative embolization in CBT surgery have been published, these reports present different and controversial results [9,10,11].

This study evaluated the complications faced in surgical resection of CBTs and the need for extended surgery, such as carotid artery resection and reconstruction. We also analyzed the relationship between surgical problems and clinical features of the patients.

## 2. Patients and Methods

### 2.1. Patients and Design

We performed our study according to the ethical standards of the responsible committee on human experimentation (institutional and national) and with the Declaration of Helsinki of 1975, as revised in 2008. This study was approved by our Institutional Review Board (Iwate Medical University Hospital Institutional Review Board CRB2210002). Informed consent was obtained from all the patients included in this study (HGH27-28). The study design was a retrospective review of patient medical records.

From March 2013 to August 2020, 20 consecutive patients with 21 CBTs were referred to our hospital and treated. There was a family history of paraganglioma in four patients. Bilateral CBTs were observed in two of these four patients. In our policy, patients with CBT should undergo resection surgery if they have no distant metastasis.

### 2.2. Preoperative Embolization

An interventional radiologist performed pre-operative embolization of the tumor-feeding arteries in all patients on the same day CBT surgery was performed. We have previously reported a detailed description of the method used for pre-operative embolization in detail [12]. Super-selective catheterization was performed in the CBT-feeding arteries. Embolization was performed repeatedly to achieve successive reduction of blood supply to the tumor.

### 2.3. Surgical Resection

After pre-operative embolization, we routinely performed surgical resection within 3 h, i.e., in the afternoon of the same day. General anesthesia was administered during the surgery. The surgical procedure has been described previously [13]. Briefly, after dissection of the common carotid artery (CCA), the carotid bifurcation was visible. Subsequent careful dissection of the internal carotid artery (ICA) and external carotid artery (ECA) resulted in complete resection of CBT. However, if dissection of the carotid artery, especially the ICA, was difficult because of firm attachment or tumor invasion of the carotid artery wall, the carotid artery was resected and reconstructed. We carefully preserved all the cranial (IX, X, XI, XII) and sympathetic nerves during surgery.

### 2.4. Tumor Size

Tumor size was measured using pre-operative computed tomography (CT) and/or magnetic resonance imaging (MRI). In this study, tumor size was represented by the maximum diameter.

### 2.5. Germline Mutation Analysis

We performed genetic testing for succinate dehydrogenase subunit B (SDHB) and succinate dehydrogenase subunit D (SDHD), after receiving written informed consent from the patients. Other genetic testing, for the genes causing paraganglioma such as succinate dehydrogenase subunit C (SDHC), von Hippel Lindau (VHL), Rearranged during transfection (RET), etc., was also conducted on the samples that were negative for SDHB and SDHD tests. Germline DNA was extracted from peripheral blood and screened using the polymerase chain reaction-directed sequencing method, as described previously [14,15,16].

### 2.6. Statistical Analyses

Statistical analyses were performed by a biological statistician (FT) using the Wilcoxon rank-sum test, Welch’s t-test, and Fisher’s exact test to determine the significant differences between the background factors of the patients and the postoperative complication and carotid artery resection. A *p* value < 0.05 was considered statistically significant.

## 3. Results

Table 1 shows the patient characteristics. This study enrolled 12 women and 8 men, with median age of 46 years (range: 18–62 years). One patient had a Shamblin type I tumor (Case 7) and one patient with type III tumor (Case 19); the other 19 had type II tumors, including two patients with bilateral CBTs (Cases 11 and 12). There were five patients with the germline mutation SDHD (Cases 2, 11, 12, 14, 15, and 18) and four patients with the germline mutation SDHB (Cases 1, 8, 9, and 20).

**Figure 1:**
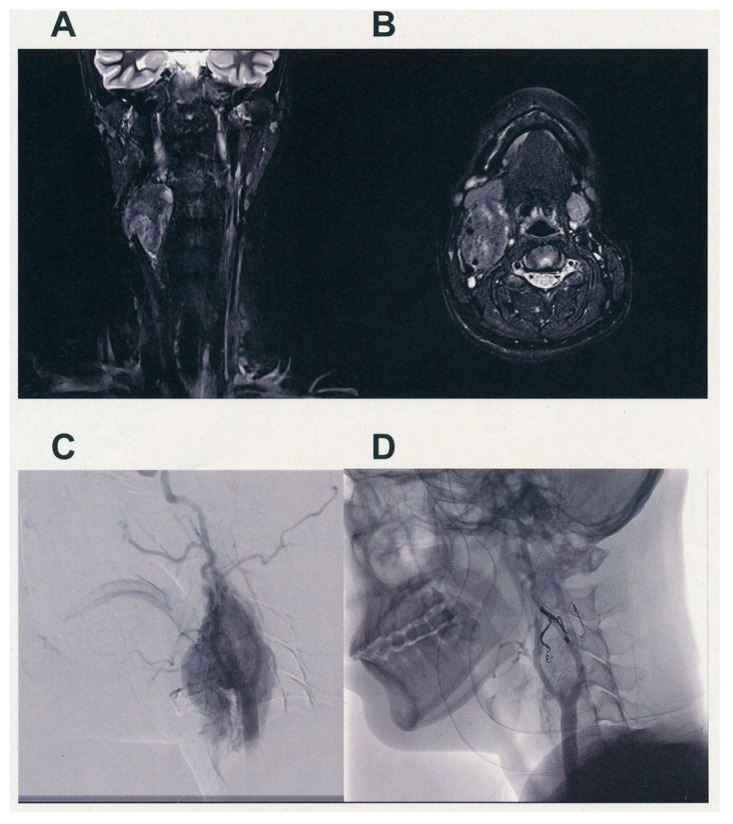
Typical diagnostic images and results of pre-operative embolization followed by surgery (Case 19). (**A**) Anterior coronal view (**B**) Axial view of T2-weighed magnetic resonance (MR) image of the carotid body tumor (CBT). The tumor is located at the right carotid bifurcation, with an estimated size of 48 × 46 × 32 mm on the image. The tumor was categorized as Shamblin type III, as it is found enclosing the right carotid bifurcation, ECA, and ICA. (**C**) Lateral view of the digital subtraction angiography (DSA) image before embolization of the feeding arteries. (**D**) The same view as in (**C**) after embolization. The feeding arteries from the ascending pharyngeal, occipital, superior thyroid, facial, and inferior thyroid arteries were selectively injected with gelatin sponge or coil embolization was performed (see Materials and Methods). Almost all blood supplies were obstructed by embolization.

**Figure 2:**
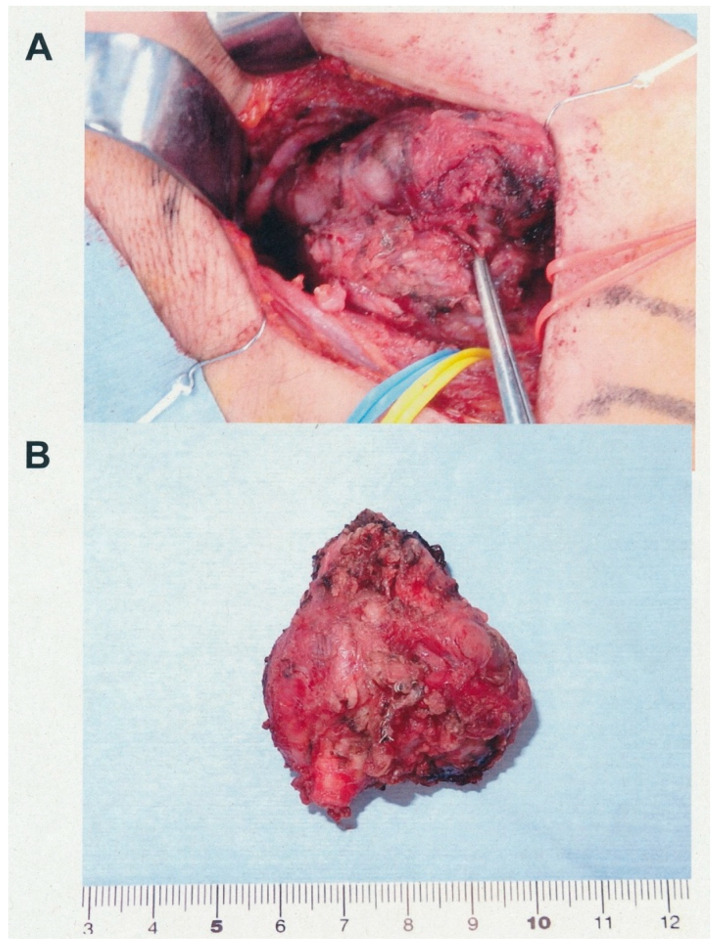
Operative view and resected specimen. (**A**) Operative view of carotid body tumor (CBT) surgical resection. The tumor covers the internal and external carotid arteries, which are not visible. The common carotid artery (red), vagal nerve (yellow), and internal jugular vein (blue) were secured using a vessel loop and preserved. The hypoglossal nerve that is visible was preserved. (**B**) Lateral view of the removed specimen. The carotid bifurcation, including the common carotid artery, internal carotid artery, and external carotid artery, were resected along with the tumor. The cut lumen of the common carotid artery is visible on the lower side of the tumor.

Figure 1 shows the pre-operative findings of the diagnostic images of case 19. There was a tumor with a pre-operative diameter of 48 mm in the right neck. The Shamblin classification of the tumor was classified as type III. Since her CBT had feeding arteries from the right ascending pharyngeal, occipital, facial, superior thyroid, inferior thyroid, and left superior thyroid arteries, pre-operative embolization was performed in the morning, followed by CBT resection in the afternoon. Our radiologist performed complete embolization of the feeding arteries, which resulted in a significant and adequate reduction in blood supply (Figure 1D). We preserved all cranial nerves safely during surgical resection; however, the tumor was firmly attached and had invaded the carotid artery wall. Therefore, a carotid artery resection followed by reconstruction was performed (Figure 2). The blood loss and operating time were 90 mL and 349 min, respectively.

Table 1 shows the results of our CBT surgeries and their respective outcomes. The mean blood loss and operative time were 39.7 ± 78.2 mL and 182 ± 112 min, respectively. The median blood loss and operative time were 9 mL and 125 min, respectively. The blood loss and operative time of the four patients (Cases 4, 17, 19, and 21), who underwent resection of the carotid artery followed by reconstruction with an artificial vessel or autologous vessel graft, were 341, 137, 90, and 97 mL and 300, 485, 349 and 440 min, respectively. Exclusion of these four cases yielded a mean blood loss and operative time of 8.0 mL and 129 min, respectively.

The feeding arteries of the patients are presented in Table 1. The ascending pharyngeal artery (APA) was the most predominant feeding artery, which fed 20 of the 21 tumors. Followed by the APA, the superior thyroid artery (STA) and the occipital artery (OA) fed 16 and 11 tumors, respectively. Surprisingly, nine tumors were fed by a direct feeder from the ECA; moreover, two tumors were fed by two direct feeders and one tumor by three direct feeders from the ECA. Additionally, the accessory superior thyroid artery fed two CBTs. Since these arteries are not found in normal individuals, they were thought to be aberrant. Surprisingly, one tumor was fed by a branch from the vertebral artery, and one was fed by the contralateral STA (Case 19). There were three tumors with six feeding arteries, five with five feeding arteries, five with four feeding arteries, four with three feeding arteries, two with two feeding arteries, and one with only one feeding artery.

Table 1 shows the post-operative complications. The most frequent post-operative complication was cranial nerve X paralysis (*n* = 9), leading to recurrent nerve palsy and hoarseness, followed by cranial nerve XII paralysis (*n* = 7). First-bite syndrome and cranial nerve IX paralysis were also observed in three independent patients. Most of the cases were transient and resolved within several days-to-months, post-operatively. One patient (Case 8) presented with transient Horner’s syndrome. Three patients (Cases 4, 17, and 19) who underwent carotid artery resection and reconstruction showed paralysis of cranial nerves IX, X, and XII post-operatively, which resolved within weeks.

We examined the relationship between the frequency of complications and patient clinical data (Table 2). Although age, tumor diameter, blood loss, SDH variants, number of feeding arteries, and presence of aberrant feeding arteries were not significant factors for the occurrence of complications, operative time was a significant factor.

We also examined the relationship between carotid artery resection and patient clinical data (Table 3). Although tumor diameter, SDH variants, number of feeding arteries, and presence of aberrant feeding arteries were not significant factors for the occurrence of carotid artery resection; blood loss, operative time, and age were significant factors. As increased blood loss and operative time were due to the technical consequences of carotid artery resection, age was the only significant factor related to the resection of the carotid artery in patients with CBT.

## 4. Discussion

Increased blood loss and long operative times due to the difficulties in dissecting a CBT from the carotid artery walls because of hypervascular capsules of the tumor has been a matter of concern for surgeons performing CBT resection surgery [17,18,19]. We developed a novel method of CBT surgery, entitled “same-day operation” which consisted of embolization of the feeding arteries in the morning and resection of CBT in the afternoon of the same day. This procedure markedly reduces the blood loss and operative time of CBT surgery [13]. However, some patients required carotid artery resection and reconstruction, owing to firm the attachment or invasion of the carotid artery wall by the tumor, resulting in increased blood loss and operation time.

Regarding blood supply, most tumors had multiple feeding arteries, with aberrant arteries originating directly from the ECA. These arterial feedings may have led to blood loss during CBT resection. In fact, 9 of the 21 tumors in our cases had direct-feeding arteries from the ECA, and there were two tumors with an aberrant accessory STA. In addition, one CBT was fed by the vertebral artery, and the other was fed by the contralateral STA. These aberrant arteries may be a consequence of slow-growing tumors that originate during the fetal period.

Analysis of the clinical characteristics of the patients showed that increased blood loss and operative time were significantly associated with increased post-operative complications such as XII or X paralysis.

Our analysis also revealed that carotid artery resection and reconstruction were closely related to the young age of the patients. The four patients who underwent carotid artery resection and reconstruction in our series were between 18 to 23 years of age. The reason why they had undergone resection of the carotid arteries must have been the firm attachment of their tumors to their arterial walls. However, there have been neither explanations nor reports why young patients’ tumors attach firmly or invad to their arterial walls.

CBT may be a sex hormone-dependent tumor, and patients around 20 years of age actively secrete these hormones. Androgen [20] and estrogen [21] are reportedly involved in angiogenesis. Endothelial cells have androgen and estrogen receptors, and can also generate capillary vessels. For example, androgens amplify tumor vessels through vascular endothelial growth factor (VEGF) interaction in prostate cancer [22] and cutaneous neurofibroma [23]. Although the role of androgen receptors in the modulation of vascular cells is evident, the molecular mechanisms by which androgen receptors regulates vascular homeostasis and disease processes are not fully understood [20]. These findings were supported by an animal model in which endothelial cells from androgen-receptor knockout mice showed attenuated angiogenic potency [24].

Vascular cells are well known to express estrogen receptors. The expression of estrogen receptors has also been reported in endothelial cells [25,26] and vascular smooth muscle cells [27,28]. In contrast, estrogen mobilizes bone marrow-derived endothelial progenitor cells, resulting in increased neovascularization of mammary tumor tissue in mice [29]. Moreover, anti-estrogens, such as tamoxifen, are effective inhibitors of angiogenesis [30]. These findings indicate that estrogen receptors in breast cancers produce tumor vessels and progress to estrogen receptor-positive tumors. The neovascularization of CBT is likely driven by sexual hormones, such as androgen and estrogen. These hormones may enhance the ability of these tumors to invade the carotid arterial wall.

Further study will be required to reveal the relationship between the younger age and the firm attachment of their CBTs to their arterial walls.

## 5. Conclusions

We proposed that a young age may influence the difficulties faced in CBT surgery, based on the results of statistical analyses, that results in an increased risk of carotid artery resection. The results obtained from our study could help surgeons safely and effectively perform resection surgery for CBTs.

## Figures and Tables

**Table 1:** Patient characteristics.

No.	Age	Gender	Side	Shamblin Type	Diameter of Tumor (mm)	Operative Time (min)	Blood Loss (mL)	Complications	SDH Variants	Feeding Arteries	Number of Feeding Arteries	Aberrant Arteries	Resection of Carotid Artery
1	31	F	R	II	31	113	16	(+)	(+)	APA, OA, APalatA, STA	4		
2	30	M	R	II	22	99	3	(−)	(+)	APA, OA, STA, ECA	4	(+)	
3	60	F	R	II	44	129	8	(+)	(−)	APA, STA,	2		
4	23	F	R	II	34	300	341	(+)	(−)	APA, STA, OA, LA, ECA	5	(+)	(+)
5	57	F	L	II	36	110	16	(−)	(−)	APA, OA, STA, PAA, LA	5		
6	45	F	L	II	38	115	12	(+)	(−)	APA, STA, LA, ECA	4	(+)	
7	42	F	R	I	22	113	5	(−)	(−)	APA, OA,	2		
8	62	F	R	II	47	138	14	(+)	(+)	APA, OA, LA	3		
9	40	F	R	II	38	165	7	(+)	(+)	APalatA, FA, STA,	3		
10	56	M	L	II	44	118	13	(−)	(−)	APA, STA, LA, ECA	4	(+)	
11	53	M	L	II	45	125	11	(−)	(+)	APA, STA, ECA	3	(+)	
12	53	M	R	II	44	161	5	(+)	(−)	APA, OA, aSTA, ECA	4	(+)	
13	57	M	L	II	29	121	9	(+)	(−)	APA, STA, ECA	3	(+)	
14	47	M	R	II	26	110	2	(+)	(+)	APA, STA, aSTA, OA, ECA	5	(+)	
15	61	M	R	II	40	179	5	(+)	(+)	APA, OA, STA	3		
16	57	F	L	II	33	105	2	(−)	(−)	APA	1		
17	20	M	L	II	47	485	137	(+)	(−)	APA, STA, ECAx3, VA	6	(+)	(+)
18	46	M	L	II	37	135	5	(+)	(+)	APA, STA, FA, LA, ECAx2	6	(+)	
19	18	F	R	III	48	349	90	(+)	n.t.	OA, FA, APA, STA, ITA, clSTA	6		(+)
20	32	F	L	II	40	146	11	(+)	(+)	APA, STA, APalatA, ECA	5	(+)	
21	18	F	R	II	30	440	97	(+)	n.t.	APA, OA, LA, ECAx2	5	(+)	(+)

SDH, succinate dehydrogenase; fa, feeding artery; M, male; F, female; R, right; L, left; APA, ascending pharyngeal artery; OA, occipital artery; STA, superior thyroid artery; ECA, external carotid artery; PAA, posterior auricular artery; LA, lingual artery; ApalatA, ascending palatine artery; FA, facial artery; aSTA, accessory superior thyroid artery; VA, vertebral artery; ITA, inferior thyroid artery; clSTA, contralateral superior thyroid artery; n.t., not tested.

**Table 2:** Analyses of the relationship between clinical factors and complications of surgery.

Characteristics	Complication (−)		Complication (+)		Wilcoxo Rank-Sum Test	*p* Value	Fisher’s Exact Test
	Median (Q1–Q3)	Mean ± SD	Median (Q1–Q3)	Mean ± SD		Welch *t*-Test	
No. of patients	6		15				
Age	54.5 (44.8–56.8)	49.2 ± 11.0	45.0 (27.0–55.0)	40.9 ± 16.2	0.4353	0.1978	
Tumor diameter	34.5 (24.8–42.0)	33.7 ± 10.1	38.0 (32.5–44.0)	38.1 ± 6.9	0.3292	0.3621	
Operative time	111.5 (106.2–116.8)	111.7 ± 9.2	146.0 (125.0–239.5)	205.7 ± 125	0.0057	0.0115	
Blood loss	8.0 (3.5–12.5)	8.3 ± 5.8	11.0 (6.0–53.0)	50.6 ± 90.7	0.2912	0.0937	
Feeding arteries	3.5 (2.3–4.0)	3.5 ± 1.5	4.0 (3.0–5.0)	4.3 ± 1.3	0.1522	0.1464	
SDH variants	2/6		7/13				0.6285
Aberrant arteries	3/6		9/15				1

**Table 3:** Analyses of the relationship between clinical factors and carotid artery resection.

Characteristics	CA Resection (−)		CA Resection (+)		Wilcoxon Rank-Sum Test	*p* Value	Fisher’s Exact Test
	Median (Q1–Q3)/Frequency	Mean ± SD	Median (Q1–Q3)/Frequency	Mean ± SD		Welch *t*-Test	
No. of patients	17		4				
Age	53.0 (42.0–57.0)	48.8 ± 10.7	19.0 (18.0–20.8)	19.8 ± 2.4	0.0026	<0.0001	
Tumor diameter	38.0 (31.0–44.0)	36.2 ± 7.9	40.0 (33.0–46.3)	39.3 ± 8.5	0.4725	0.5525	
Operative time	121.0 (113.0–138.0)	128.4 ± 22.8	393.5 (336.8–451.2)	393.5 ± 84.2	0.0027	0.0075	
Blood loss	8.0 (5.0–12.0)	8.5 ± 4.7	117.0 (92.3–188.0)	166.3 ± 118.3	0.0026	0.0759	
Feeding arteries	4.0 (3.0–4.0)	3.6 ± 1.3	5.5 (5.0–6.0)	5.5 ± 0.6	0.0119	0.0009	
SDH variants	8/17		2/2				0.4737
Aberrant arteries	8/17		1/4				0.6030

## Data Availability

The data presented in this study are available on request from the corresponding author. The data are not publicly available due to privacy or ethical restrictions.
